# Incidence and predictors of loss to follow-up among adult HIV-infected patients taking antiretroviral therapy at North Shewa zone public Hospitals, Northeast Ethiopia: A retrospective follow-up study

**DOI:** 10.4314/ahs.v22i2.3

**Published:** 2022-06

**Authors:** Wondimeneh Shibabaw Shiferaw, Abebe Muche Belete, Alemu Adela, Mekasha Getnet, Yared Asmare Aynalem

**Affiliations:** 1 Department of Nursing, College of Health Science, Debre Berhan University, Ethiopia; 2 Department pre-clinical, College of Medicine, Debre Berhan University, Ethiopia

**Keywords:** ART, HIV/AIDS, lost to follow up, predictors, Ethiopia

## Abstract

**Background:**

Patients who are lost to follow-up while on treatment compromise their own health and the long-term success of antiretroviral therapy (ART) programs. Besides, loss to follow-up (LTFU) increases HIV-related morbidity and mortality. Therefore, this study aimed to determine the incidence of LTFU and its predictors among adult HIV positive patients on anti-retroviral therapy at North Shoa zone public hospitals, Northeast Ethiopia.

**Methods:**

A retrospective follow up study of 517 people living with HIV/AIDS and attending an ART clinic between 2015 and 2020 was conducted at North Shewa zone, public hospitals. Kaplan-Meier failure function together with log rank test was used to compare failure function. Multivariable Cox proportion hazards regression model was used to determine predictors of LTFU.

**Result:**

The incidence density rate of lost to follow up among HIV positive adult on ART was found to be 8.9 per 100 adult years observation (95%CI; 7.45, 10.68). In multivariable cox proportional regression analysis, WHO clinical stage-IV (AHR = 1.50; 95% CI: 1.08, 3.75), comorbidity disease (AHR = 0.54; 95% CI; 0.30, 0.97), body mass index less than 18kg/m2 (AHR = 1.60; 95% CI; 1.02, 2.51), cotrimoxazole preventive therapy (AHR = 1.57; 95% CI;1.09, 2.53), and a low CD4 count (AHR = 1.66; 95% CI; 1.29, 3.49) were found to be a significant predictors of lost to follow up.

**Conclusion:**

The current study showed that the incidence rate of loss to ART follow-up was high. Body mass index score less than 18kg/m2, advanced WHO clinical stage, CD4<200cell/mm^3^, had comorbidity disease, and cotrimoxazole therapy were a significant predictors of lost to ART follow up. Therefore, appropriate mitigation measures in the at-risk group need to be instigated to advance retention rate.

## Background

Human Immunodeficiency Virus (HIV) has been a global challenge for the past three decades. In 2017, nearly 36.9 million People living with HIV (PLHIV) worldwide, of these 35.1 million were adults, and about 19.6 million are in Eastern and southern Africa making up 53.1% of PLHIV in the region^1^. Sub-Saharan Africa (SSA) carries the highest burden with an estimated 24.7 million PLHIV, nearly 71% of the global total^2^. There has been an increasing in number of PLHIV in Ethiopia, estimated number of PLHIV were 710,000 people are still infected, with adult greater than 15 years, and the estimated adult prevalence making up 1.1%^3^, ^4^.

Evidence showed that ART significantly reduced mortality and improved life expectancy of HIV-infected patients, but the attainment depends on regular patient follow-up^5^. The goals of ART are maximal and durable suppression of viral replication to prevent development of HIV drug resistance and treatment failure, restoration and/or preservation of immunologic function, reduction of HIV-related morbidity and mortality, improving the quality of life^6^. As ART coverage expands, a rise in loss to follow-up (LTFU) has been observed in many ART programs in Sub-Saharan Africa^7^.

The high levels of adherence to ART and retention in HIV care programs is critical to achieve optimal health outcomes among PLHIV^8^. However, LTFU has a significant clinical and epidemiological challenge, which compromises long term patients' survival^9^. Besides, it negatively impacts on the immunological benefit of ART and increases AIDS-related morbidity, mortality, and hospitalizations^10^. Moreover, it would result in serious consequences, such as drug toxicity, treatment failure, and drug resistance^11^–^13^.

Several studies showed that the predictors of lost to follow up were daily laborer^14^, ambulatory functional status^14^–^17^, not take opportunistic prophylaxis^14^, ^15^, ^17^, ^18^, being underweight 15, 19, 20, jobless^15^, substance abuser 15, having sub-optimal adherence 15–17, having opportunistic infections^15^, having CD4 count <200 cells/µL^15^, ^17^, ^18^, old age 20, 21, female gender 16, 22, and advanced disease stage^17^–^19^. The incidence of LTFU was estimated to be 103 per 1000 person-years in South Africa 7, 10 per 100 in Zambia^23^, 52.9 per 100 person-years in Kenya 24, 11.6 per 100 person-years in northwest Ethiopia 25. Although previous studies have explored risk factors associated with LTFU from ART programs, the problem is highest remains a key challenge for program planners. Therefore, it needs further exploration of predictors of LTFU. Besides, there was limited evidence on the incidence of LTFU and its predictors in the study area. Hence, this study aimed to estimate the incidence of LTFU and its predictors among HIV positive adult patients on ART at North Shewa zone public hospitals, Northeast Ethiopia. Findings will be important evidence to know burden of the problem to develop management and preventive measures.

## Methods

### Study setting

The study was conducted in North Shewa Zone, public hospitals, Northeast Ethiopia. North Shewa Zone is one of the 13 Zones in Amhara administrative regional state and the City is Debre Berhan Town located, 695 km from Bahir Dar, capital of the region and 130 km from Addis Ababa, capital of Ethiopia. It is border on the south and the west by the Oromia region, on the North by South Wollo, on the North East by the Oromia zone, and the East by the Afar region. It covers an area of 15,936.13 square kilometers^26^. It has 27 districts with different climate (dega, woyneadega & kola). The major ethnic groups of the zone are Amhara. The population is predominantly Orthodox-Christian by religion followed by Muslim. Agriculture is the main livelihood of the pop-ulation^26^. The Zone has 11 hospitals (10 of which are public). Out of the 10 public hospitals, two are general hospitals; one is comprehensive specialized hospital and the rest 7 are primary hospitals. According to North Shoa zonal health office report of 2017 for comprehensive ART services (HIV/AIDS care) in all health facilities, on these selected hospitals the total of 3406 HIV-positive adult patients were received clinical care. The study was conducted among the selected four public hospitals (Debre Berhan Comprehensive Specialized Hospital, Enat Primary Hospital, Mehal Meda Primary Hospital, and Ataye Primary Hospital).

### Study design and population

A five-year institution based, retrospective follow up study was conducted at North Shewa zone public hospitals from February 1 to March 15, 2020. All adult HIV/AIDS patients who were newly initiated ART and enrolled at selected north shoa zone public hospitals, ART clinic during the required time (i.e. January 2015 to January 2020) was considered in the current study. Besides, clients who had diagnosis and initiated ART at other health institution and subsequently referred to the respective selected hospital for further treatment was included. On the other hands, HIV positive adult patients, whose medical charts were not found and having incomplete data were excluded from the study.

### Sample size, sampling technique, and covariates

The sample size was determined using double population proportion formula by considering WHO clinical stage as the major predictor variables^25^ using Epi info version 7 statistical package. Based on the available evidence the final sample size determined for this study was 517 HIV/AIDS patients' charts. The estimated sample size was proportionally allocated to each selected four hospitals according to their total population and the data were collected by systematic random sampling. The sampling intervals of the hospitals are different which was determined by dividing the total number of ART patients chart in the specific hospital to the allocated sample size (N/n) th. The first patient medical chart was selected randomly from the registration book and every K patient chart for all selected hospital was selected. The incidence of lost to follow up among HIV positive adult patients on ART was the outcome variable. On the other hand, age, gender, residence, body mass index, opportunistic infection, functional status, HAART regimen, WHO clinical stage, CD4 count, contact person, disclosure status, and cotrimoxazole preventive therapy were the independent variables.

### Data collection tool

A data abstraction format was develop from the standardized ART entry and follow up form currently used by the ART clinics. The data were extracted by reviewing patients ART medical charts. Lost to follow up was confirmed by reviewing medical registration in each hospital. The data abstraction format was examined by senior experts to the area of study for content validity. Data abstraction was designed based on study objectives. This included socio-demographic characteristics, baseline clinical information, and treatment outcomes were obtained from patients' medical charts. These clinical and treatment related predictors were taken in to consideration at starting of ART was used as a baseline value. Data were retrieved from patients' ART chart by trained nurses working in the ART clinic using uniform data abstraction format prepared for the study.

### Data Collection Procedure

All available information on patient records were checked then appropriate data extraction format was adopted in English in order to extract all the relevant variables to meet the study objectives. The starting point for retrospective follow-up was the time from first initiation of ART among HIV positive adult patients and the endpoint was date of lost to follow up, death, transfer, date of last contact until January 1st, 2020 which ever come first. The chart of HIV positive adult patients enrolled on ART from January 2015 to January 1^st^, 2020 at selected four hospitals was retrieved from ART registries. The record of all study participants was selected according pre-specified eligibility criteria. Time to lost to follow up was calculated as the time between the date of ART initiation and the date of lost to follow up. In the current study, HIV positive patients were considered as censored, if transfer to other health care institution (based on reported date or last visit date) or if they were alive at the end of the study period (January 1^st^, 2020). Before collecting the data, baseline and follow up records were reviewed and the registration was identified by their medical record number. The data collectors who are working at the ART clinic were extracted and reviewed the charts.

### Data Quality Control

To ensure the quality of the data pretest was conducted with 5% of the data abstraction format, to check the hospital documentation system with the agreement of the data abstraction format to the objective of the study. Any error found during the process of checking was correct and modification was made into the final version of the data abstraction format. Training on record review was given to data collectors and supervisors for 01 days before actual data collection task. Besides, the data quality was controlled by designing the proper data collection materials, and through continues supervision. The collected data form was examined for completeness and consistency during data entry, storage, cleaning and analysis. The principal investigator of the study was control the overall activity.

### Data Analysis

Data were coded, cleaned, and entered using EPI-data 3.1 and exported to STATA 14 statistical software for analysis. Kaplan-Meier failure function together with log rank test was used to test for the presence of difference in lost to follow up among categories of covariates. Appropriate descriptive statistics were used based on the nature of the data. Bivariable cox regression analysis was first fitted and those independent variables, with a p-value ≤ 0.25 level of significance, were included in the multivariable analysis. Multivariable cox proportional-hazard regression was fitted at 5% level of significance to determine the net effect of each independent variable on lost to follow up after initiation of ART among HIV positive adult patients. To verify the proportionality assumption in Cox's model, we tested the correlation between survival time and Shoenfeld's standardized residuals. The result of this study was expressed as hazard ratios (HRs) with 95% confidence interval. Finally, the result of the study was presented with text, graph and table.

## Result

### Socio-demographic characteristics of the study participants

The median age of the respondents was 34 (IQR: 28–40 years) and nearly than half (41 %) of them were under the age category of 25–34 years. With respect to sex of the participants about 265(52.27%) were male. Majority, 347(68.44%) of the participants were urban by residence. Nearly half, 240(47.34%) of the participants were married. Majority, 392(77.32%) of the respondents are orthodox religion follower. One hundred seventy two (33.93%) of the respondents have no formal education and only 20.2% have higher educational level. Regarding occupational status nearly one third, 144(28.4%) has self-employee work. Most of them, 410 (80.87%) have contact person during their follow up care (Table 1).

**Table 1:** Socio-demographic characteristics HIV/AIDS patients on ART

Variable	Category	Frequency	Percent (%)
Sex	Male	265	52.27
	Female	242	47.73
Age	15–24	59	11.64
	25–34	208	41.03
	35–44	151	29.78
	>45	89	17.55
Residence	Rural	160	31.56
	Urban	347	68.44
Marital status	Married	240	47.34
	Single	102	20.12
	Divorced	125	24.65
	Widowed	40	7.89
Religion	Orthodox	392	77.32
	Muslim	87	17.16
	Other*	28	5.52
Occupation	Daily labor	64	12.62
	Farmer	88	17.36
	Government employee	107	21.10
	Self-employee	144	28.40
	Student	27	5.33
	House wife	77	15.19
Educational status	No formal education	172	33.93
	Primary school	136	26.82
	Secondary school	97	19.13
	Higher education	102	20.12
Contact person	Yes	410	80.87
	No	97	19.13

**NB**: other*; catholic and protestant

### Clinical related characteristics

Nearly one third, 158 (31.16%), had opportunistic infections, of these 86 (54.43%) had Tuberculosis, 32 (20.25%) bacterial pneumonia, and 16 (10.13%) other opportunistic infections. Nearly one quarter, 122(24.06%) had comorbidity disease at baseline. Regarding body mass index more than one quarter, 138(27.22%) had less than 18.5 kg/m^2^. The majority of the participants, 379 (74.75%), were at working functional status. Regarding the WHO clinical stage over one third 194(38.26%) were clinical stage I, while one third, 166 (32.74%) were clinical stage III. Majority, 411(81.07%) of the study participants were disclosed their status to anyone (Table 2).

**Table 2:** Clinical characteristics of HIV/AIDS patients on ART

Variables	Category	Frequency	Percent (%)
Opportunistic infection	Yes	158	31.16
	No	349	68.84
Type of Opportunistic infection	Herpes zoster	7	4.43
	Pneumonia	32	20.25
	Tuberculosis	86	54.43
	Oral thrush	17	10.76
	Other*	16	10.13
Comorbidity	Yes	122	24.06
	No	385	75.94
Body mass index	< 18.5	138	27.22
	18.5–24.9	328	64.69
	≥. 25.0	41	8.09
functional status	Working	379	74.75
	Ambulatory	115	22.68
	Bedridden	13	2.56
Hemoglobin(mg/dl)	Anemic	96	18.93
	Normal	411	81.07
WHO clinical staging	Stage I	194	38.26
	Stage II	127	25.05
	Stage III	166	32.74
	Stage IV	20	3.94
Baseline CD4	<200	125	24.65
	200–500	337	66.47
	>500	45	8.88
Disclosure status	Disclosed to any one	411	81.07
	Not disclosed to any one	96	18.93

NB: Other*

### Treatment related characteristics

Nearly one third, 156 (30.77%) of the patients were eligible for ART by WHO clinical stage and total lymphocyte count, while 151 (29.78%) were so by CD4 cell count criteria. Cotrimoxazole Preventive Therapy (CPT) was given to 338 (66.67%) study participants. More than half, 278 (54.83%), of the patients started ART treatment with TDF–3TC-EFV and 36 (6%) changed to another regimen within the first line, and while 111(21.89) were used AZT-3TC-NVP regimens. One fourth, 132 (26.04%) of study participants had reported drug side effect (Table 3).

**Table 3:** Treatment related characteristics of participants among HIV/AIDS patients on ART

Variable	Category	Frequency	Percent
ART eligibility criteria	CD4<350, 500	151	29.78
	WHO stage IV	14	2.76
	WHO stage II and III with TLC<120	156	30.77
	No criteria	186	36.69
HAART regimens given	1c(AZT-3TC-NVP)	111	21.89
	1d(AZT-3TC-EFV)	45	8.87
	1e(TDF-3TC-EFV)	278	54.83
	1f(TDF-3TC-NVP)	36	7.10
	Others*	37	7.29
Cotrimoxazole prophylaxis	Given	338	66.67
	Not given	169	33.33
Drug side effect	Yes	132	26.04
	No	375	73.96
Last follow up status	Lost to follow up	118	23.27
	Alive	363	71.59
	Death	18	3.55
	Transfer	8	1.58

**NB**: others*; 1J (TDF+3TC+DTG), and 2h (TDF+3TC+ATV/r)

### Incidence of lost to follow up

**Figure 1:**
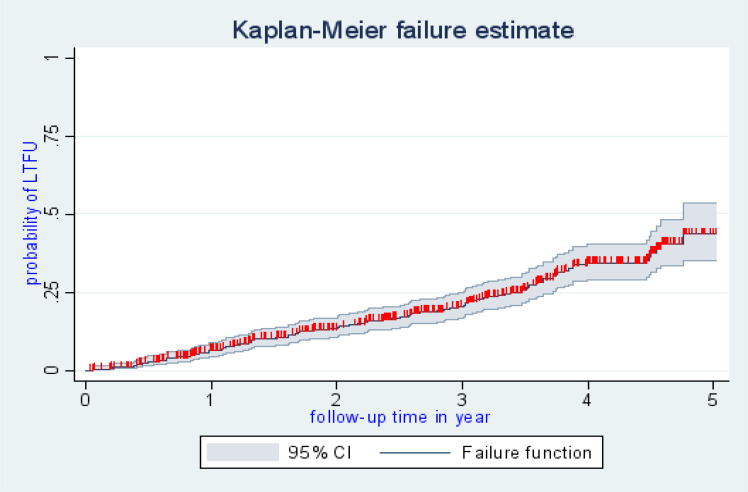
The overall hazard rate of lost to ART follow-up among HIV-positive adult individuals at North Shoa, public hospitals, Northeast Ethiopia.

**Figure 2:**
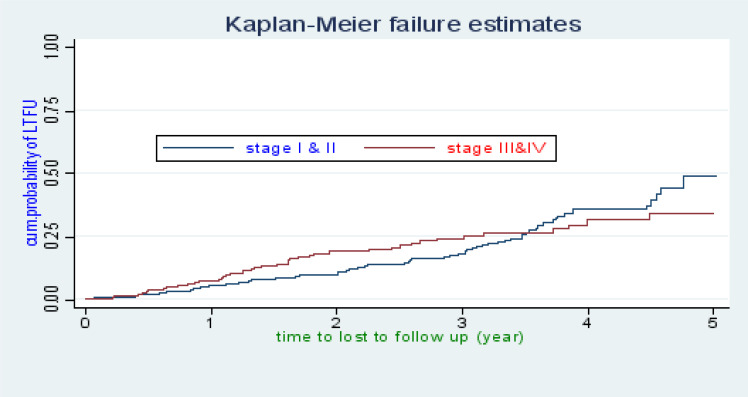
The hazard rate of lost to follow-up of HIV-positive adults on ART by their WHO clinical stage

**Figure 3:**
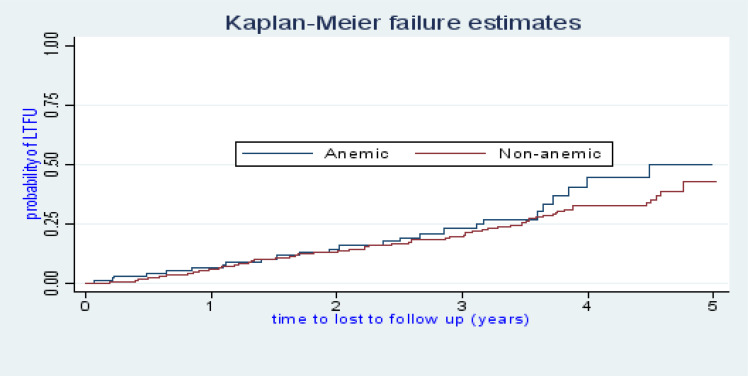
The hazard rate of lost to follow-up of HIV-positive adults on ART by their baseline anemic status

**Figure 4:**
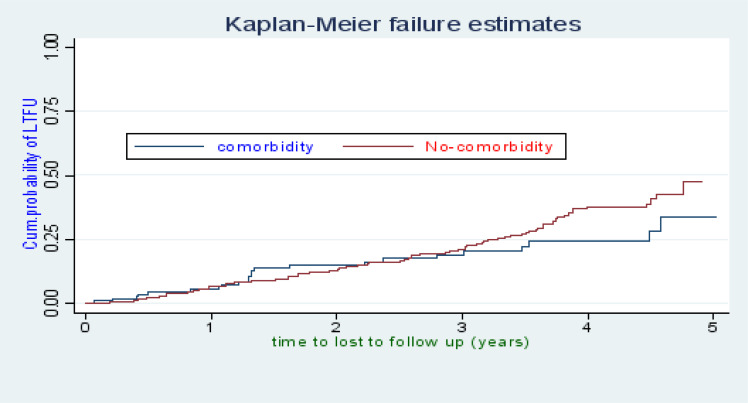
The hazard rate of lost to follow-up of HIV-positive adults on ART by their comorbidity status

**Figure 5:**
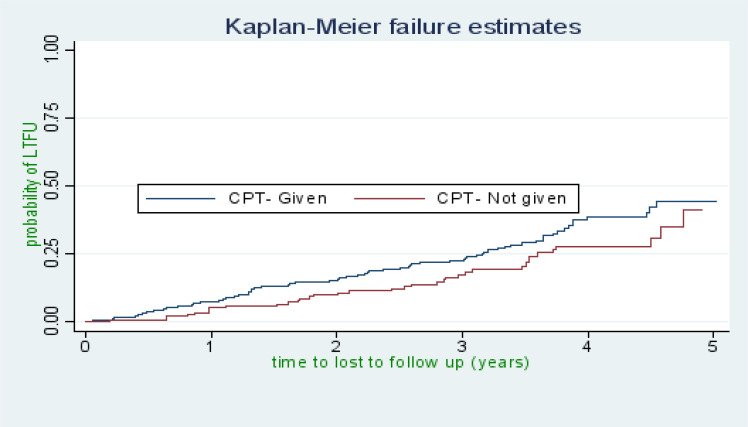
The hazard rate of lost to follow-up of HIV-positive adults on ART by their cotrimoxazole therapy

There were 118 cases of LTFU observed over 1,322 person years at risk. The incidence rate of lost to follow up was 8.9 per 100 person years observation (95%CI; 7.45, 10.68). The incidence proportion of lost to follow up in the cohort was 23.27%. The study participants were followed for a median of 32.46 months (IQR: 15.63–45.05) after initiation of ART. The probability of lost follow up among ART patients was 6.32%, 13.16%, 20.74%, 34.24%, and 43.59% at 1, 2, 3, 4, and 5 years of follow up respectively (Fig. 1). The overall lost to follow up failure rate of the cohort during 5 years follow up was 43.59%. The cumulative probability of LTFU among different groups of ART patients by their WHO clinical stage (Fig. 2), baseline anemic status (Fig.3), comorbidity (Fig.4), and cotrimoxazole therapy (Fig.5) were compared graphically.

### Testing overall model fit

**Figure 6:**
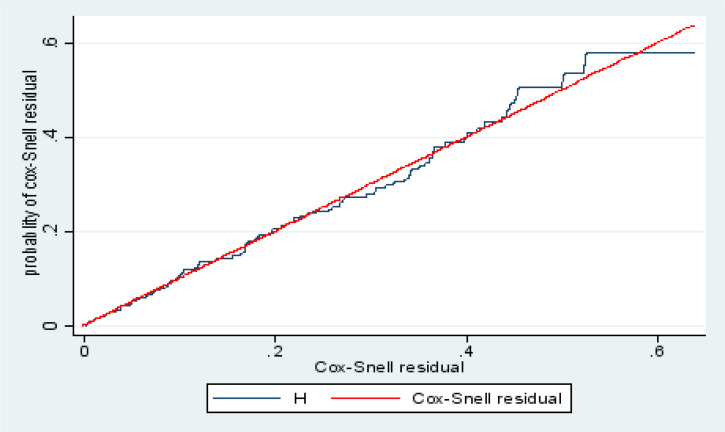
Cox-Snell residual Nelson -Aalen cumulative hazard graph on lost to follow up in North Shoa zone, public hospitals, northeast Ethiopia

This figure shows if the cox regression model fits the data, these residuals should have a standard censored exponential distribution with hazard ratio. If we comparing the jagged line with the reference line, we observe that, the cox model does fit these data to reasonable. The hazard function follows the 45° line very closely. Hence, the output shows cox-snell residuals were satisfied the overall model fitness test (Fig. 6).

### Predictor's of lost to follow up among adult ART clients using the Cox proportional regression model

Adults HIV/AIDS patients on ART who were on WHO clinical stage-IV increased the hazard ratio of LTFU by 1.5 (AHR = 1.50; 95% CI: 1.08, 3.75) times as compared to adults in clinical stages I. Having comorbidity disease at baseline were lowered the hazard ratio for LTFU by 46% (AHR = 0.54; 95% CI 0.30, 0.97) as compared to their counter parts. Body mass index less than 18k/m2 were increased the hazard ratio for LTFU by 60% (AHR = 1.60; 95% CI 1.02, 2.51) as compared with normal weight patients. Those who taken cotrimoxazole preventive therapy were increased the hazard ratio for LTFU by 57% (AHR = 1.57; 95% CI 1.09, 2.53) as compared with don't taken cotrimoxazole therapy. Adults patients who started ART with a baseline CD4 cell count of under 200 cell/m3 were 66% more likely to be LTFU compared to those with a baseline CD4 cell count of greater than 500 cell/m3 (AHR = 1.66, 95% CI, 1.29, 3.49), (Table 4).

**Table 4:** Predictor's of lost to follow up among adult ART clients at North Shewa, public hospitals, northeast Ethiopia

Variables	Category	Lost to follow up		CHR with 95%CI	AHR with 95%CI

		Yes	No		
Age in years	15–24	17	42	1	
	25–34	48	160	0.74 (0.42, 1.30)	0.72(.40, 1.29)
	35–44	34	117	0.69(.38, 1.24)	0.61(0.32, 1.16)
	>45	19	70	0.72(0.37, 1.40)	0.66(0.33, 1.33)
Contact person	Yes	93	317	1	1
	No	25	72	1.34(0.86, 2.09)	1.39(0.82, 2.36)
Opportunistic infection	No	78	271	1	1
	Yes	40	118	1.08(0.73, 1.58)	1.50(0.91, 2.46)
Comorbidity	No	95	290	1	1
	Yes	23	99	0.76(0.48, 1.20)	0.54(0.30 , 0.97)*
Baseline Body mass index	<18.5	37	101	1.31(1.08,2.14)*	1.60(1.02, 2.51)*
	18.5–24.9	76	252	1	1
	>25.0	5	36	0.57(0.23, 1.43)	0.72(0.28, 1.83)
Disclosure status	Disclosed to any one	95	316	1	
	Not disclosed to any one	23	73	1.16(0.74, 1.83)	1.19(0.69, 2.06)
Baseline CD4	<200	37	88	1.47(1.06, 2.27)*	1.66(1.29, 3.49)*
	200–500	71	266	0.83(0.42, 1.61)	0.55(0.26, 1.18)
	>500	10	35	1	1
Baseline Hemoglobin(g/dl) (logbook)	Normal	93	318	1	
	Anemia	25	71	1.24(0.79, 1.92)	1.01(0.61, 1.66)
Baseline WHO clinical stage	Stage I	44	150	1	1
	Stage II	32	95	1.12(0.71, 1.76)	0.89(0.53, 1.52)
	Stage III	35	131	0.95(0.61, 1.48)	0.82(0.47, 1.43)
	Stage IV	7	13	2.43(1.09, 5.40)*	1.50(1.08, 3.75)*
HAART regimen	1f(TDF-3TC-NVP)	7	29	1	1
	1c(AZT-3TC-NVP)	35	76	1.44(0.64, 3.26)	1.98(0.82, 4.78)
	1d(AZT-3TC-EFV)	4	41	0.40(0.12, 1.38)	0.47(0.13, 1.72)
	1e(TDF-3TC-EFV)	66	212	1.26(0.57, 2.75)	1.56(0.68, 3.61)
Cotrimoxazole Therapy	Not given	36	133	1	1
	Given	82	256	1.46(1.28, 2.16)*	1.57(1.09, 2.53)*

**NB**; *p-value <0.05 ; 1= reference group

## Discussion

This study mainly assessed the incidence and predictors of LTFU among adult HIV patients on ART in North Shewa zone, public hospitals, northeast Ethiopia. In this study the incidence rate of LTFU was found to be 8.9 per 100 person year. The finding was consistent with studies done in different regions of Ethiopia^14^, ^27^, and South Africa^28^. The observed similarity might be due to the standardized implementation of ART services based on the WHO ART guideline. However, the current finding was lower than a study done in Guinea-Bissau^29^, Uganda^30^, Colombia^31^, and different areas of Ethiopia^15^, ^32^. The possible variation among these studies could be accounted for by the different LTFU definitions for patients on ART have been proposed ranging from 60 days after a missed appointment to 180 days after the date of the last visit^33^, ^34^. Besides, the observed difference might be due to the study period in which currently, beginning from 2016, test and treat strategy is introduced in Ethiopia and this might increase the number of patients on follow up. Moreover, despite the relatively low rate of LTFU in our study, it is imperative to aim at retaining all patients on treatment since LTFU may leads to negative health outcomes such as the spread of HIV infection and deaths.

On the other hands, the present study was higher than different studies done in Uganda [20], Debre Markos-Ethiopia^35^,Yunnan province^36^, and Asia 37. Lost to follow up in our study was found to be higher than the other studies as explained above it might be due to there have been a variety of study definitions with a vast diversity of LTFU estimates makes comparisons between studies difficult. Likewise, evidence supported that the estimates of LTFU in different areas of sub-Saharan Africa are likely to be overestimated due to the absence of active tracing strate-gies^38^. Furthermore, the main reasons in rising incidence of LTFU have been attributed to poor patient tracing in the low-income setting and due to lack of reporting the risk of death events that can be considered as LTFU^39^.

According to the present study having a body mass index (BMI) score of less than 18.5 kg/m^2^ were [AHR: 1.60, 95%CI; 1.02, 2.51] found to be at higher risk of LTFU from adult ART treatment programs using 18.5–24.9 kg/m2 as reference. This finding is consistent with studies conducted in Tanzania^19^, Kenya^40^, and Northwest Ethiopia [15]. The observed similarity might be attributed to patients who were under-weight, may have had concomitant opportunistic infections related to malnutrition and some may die from such conditions but were never reported to the HIV clinic due to the passive reporting mechanism. Besides, studies showed that lower BMI have been reported to be associated with HIV mortality and progression to AIDS^41^–^43^. Furthermore, this could be due to the fact that underweight patients may have had worse baseline health and less healthy food intake to have the energy to visit frequently the clinics and keep their appointments than patients who are normal weight.

In the current study, the hazard of loss to follow among WHO clinical stage IV was 1.5 times higher as compared to clinical stage I (AHR = 1.50, 95% CI; 1.08, 3.75). A similar finding was reported by a study conducted in Tanzania^19^, in different areas of Nigeria^44^, ^45^, and Ethiopia^35^, ^46^. This could be explain by the fact that patients classified as WHO clinical stage 3 or 4 shows higher probability of infections and are bed ridden most of the time. This is likely to make their continued engagement with the HIV clinic difficult. Besides, the association between LTFU and advanced WHO clinical disease suggest that unrecognized mortality explains a proportion of LTFU among study participants^47^.

We observed that patients with baseline CD4 cell count less than 200 cells/mm^3^ were more likely to be LTFU. This finding was supported by studies conducted in Yunnan province 36, South Africa^48^, and different areas of Ethiopia 18, 46, 49. The possible explanation might be patients with a low CD4 cell count may be prone to LTFU due to significant HIV-related symptoms. Besides, patients at risk of progression could choose to live close to their family or could be transferred to another clinical center and thus be lost to follow-up in the previous center. Moreover, evidence showed that patients with low CD4 cell counts are likely to be in advanced WHO clinical stage of HIV/AIDS, which prone to opportunistic infections and an elevated risk of mortality^50^, ^51^. In contrary to previous studies^14^, ^25^, the current finding showed that patients who had been taken cotrimoxazole preventive therapy were increased the hazard ratio of LTFU. The observed difference in these studies may be due to the variation in confounding variables that were not controlled by the respective studies as it could affect the true effect of cotrimoxazole therapy on LTFU.

The current study found that the risk of LTFU among those who had comorbidity disease was reduced by 46% (AHR 0.54; 95% CI 0.30, 0.97) compared with those had no comorbidity disease. It is in accordance with the study conducted in Nigeria^30^. This might be due to the fact that the co-existence of comorbidity, patients may seek health care institution frequently. As evidence showed that comorbidity increases with HIV severity and the greater prevalence of comorbidities among people living with HIV/AIDS (PLWHA) may be attributed to antiretroviral toxicity or caused by the HIV infection itself 52. In addition, a study had done in London showed that 29% of HIV patients have at least one comorbid disease^53^. Moreover, thought HIV-non-communicable disease (NCD) comorbidity causes an additional burden to PLWHA because of increased transport costs, NCD prescribed medication expenses and more productive days lost due to illness compared with HIV alone 54.

The present study had certain limitations that should be taken into account while interpreting these findings. First, we used secondary chart review; excluding of incomplete data may leads to selection bias. Second, patients, who were considered as LTFU might have been died or self-referred to other facilities. Third, since this study used baseline socio-demographic, clinical and treatment related factors, there may be a change of these variables after a time.

## Conclusion

In the current study the incidence rate of LTFU among adult HIV/AIDS patients on ART was found to be high. Body mass index score less than 18kg/m^2^, advanced WHO clinical stage, CD4<200cell/mm^3^, had comorbidity disease, and using of cotrimoxazole therapy were statistical associated to LTFU. Therefore, policymaker and other concerned bodies should implement situation based and context specific preventive measure on these identified factors.

## Data Availability

The data used to support the findings of this study are included in the article.

## Abbreviations

AIDS: Acquired Immunodeficiency syndrome; AHR: Adjusted hazard Ratio; ART: anti-retroviral treatment: CI; Confidence Interval; CHR; Crude Hazard ratio; LTFU: lost to follow up; HIV: Human Immunodeficiency Virus; PLWHA: people living with HIV/AIDS; NCD: Non-communicable disease; WHO: World Health Organization.
